# Inflammatory Responses in Oro-Maxillofacial Region Expanded Using Anisotropic Hydrogel Tissue Expander

**DOI:** 10.3390/ma13194436

**Published:** 2020-10-06

**Authors:** Kholoud Mohamed Ali Salim, Aminah Abd Jalil, Zamri Radzi, Siti Mazlipah Ismail, Jan T. Czernuszka, Mohammad Tariqur Rahman

**Affiliations:** 1 Department of Oro-Maxillofacial Surgical & Medical Sciences, Faculty of Dentistry, University of Malaya, Kuala Lumpur 50603, Malaysia; kholoud2016kholoud@gmail.com (K.M.A.S.); sitimazlipah@um.edu.my (S.M.I.); 2 Dental Specialist Centre, Hospital Angkatan Tentera Tuanku Mizan, Kuala Lumpur 53300, Malaysia; amie_nh2@yahoo.com; 3 Department of Paediatric Dentistry & Orthodontics, Faculty of Dentistry, University of Malaya, Kuala Lumpur 50603, Malaysia; zamrir@um.edu.my; 4 Department of Material, University of Oxford, Parks Road, Oxford OX1 3PH, UK; jan.czernuszka@materials.ox.ac.uk; 5 Dean’s Office, Faculty of Dentistry, University of Malaya, Kuala Lumpur 50603, Malaysia

**Keywords:** tissue expansion, anisotropic hydrogel, biomaterials, inflammatory cytokines, cellular biology

## Abstract

Objective: Reconstruction of oral and facial defects often necessitate replacement of missing soft tissue. The purpose of tissue expanders is to grow healthy supplementary tissue under a controlled force. This study investigates the inflammatory responses associated with the force generated from the use of anisotropic hydrogel tissue expanders. Methods: Sprague Dawley rats (n = 7, body weight = 300 g ± 50 g) were grouped randomly into two groups—control (n = 3) and expanded (n = 4). Anisotropic hydrogel tissue expanders were inserted into the frontal maxillofacial region of the rats in the expanded group. The rats were sacrificed, and skin samples were harvested, fixed in formalin, and embedded in paraffin wax for histological investigation. Hematoxylin and eosin staining was performed to detect histological changes between the two groups and to investigate the inflammatory response in the expanded samples. Three inflammatory markers, namely interleukin (IL)-1α, IL-6, and tumor necrosis factor-α (TNF-α), were analyzed by immunohistochemistry. Result: IL-1-α expression was only observed in the expanded tissue samples compared to the controls. In contrast, there was no significant difference in IL-6, and TNF-α production. Histological analysis showed the absence of inflammatory response in expanded tissues, and a negative non-significant correlation (Spearman’s correlation coefficient) between IL-1-α immune-positive cells and the inflammatory cells (r = −0.500). In conclusion, tissues that are expanded and stabilized using an anisotropic self-inflating hydrogel tissue expander might be useful for tissue replacement and engraftment as the expanded tissue does not show any sign of inflammatory responses. Detection of IL-1-α in the expanded tissues warrants further investigation for its involvement without any visible inflammatory response.

## 1. Introduction

Around 20–50% of the global population are suffering from chronic periodontitis, gingival recession, and early tooth loss due to chronic inflammation of the gum. With long-term edentulism, a proper dental rehabilitation and aesthetic outcomes largely depend on the critical soft tissue [1]. Moreover, a significant amount of soft tissue is needed to seal a dental implant. Atrophy of oral mucosa following tooth loss is very common among patients, and maintenance of the epithelial and connective tissues around implants is fundamental to implant success [2]. Approximately 1 in 700 live births have a prevalence of cleft lip and palate, and 3200 new cases each year are expected. Clefts are the most common major malformation in the oro-facial region [3]. Comprehensive reconstructive treatment of facial clefts requires thoughtful consideration to obtain a proper quality and quantity of available soft tissue [4]. Additionally, proper speech is the main goal following repair of palatal function, yet it is hard to be achieved due to soft tissue tension secondary to a traditional flap [5]. Reconstruction of large facial defects following tumor excision has been challenging due to lack of local tissues and less satisfactory aesthetics outcome due to color mismatching when distant free grafts are used [6]. 

The aim of tissue expansion in reconstructive surgery is to produce a healthy supplement soft tissue under controlled mechanical tensile force for the replacement of damaged tissue [7,8]. Hydrogel tissue expanders have heralded a significant advance in clinical application [4], including reconstruction of a wide range of skin and soft tissue defects thanks to the suitable sizes for different anatomical sites. However, when tissues are expanded, tissue creep occurs [9,10]. This creep leads to changes in the intra- and extracellular environment [11,12]. Such changes provoke the release of biological signaling mediators for tissue adaptation [13,14] by activation of cellular pathways to initiate the response to the applied force [12,15].

Several mediators are known to be involved during such a process, for example, enzymes, hormones, neurotransmitters, and cytokines [16]. Cellular signaling pathways [17,18] and mechanotransduction pathways [9] start a cascade phenomenon resulting in cellular growth and new vascular formation [9]. However, it is still unclear how in vivo inflammatory responses would take place under mechanical strain. A good number of studies focused on the clinical application of the hydrogel tissue expander and the molecular investigation on the cellular changes. However, studies on the presence of inflammatory cells and inflammatory cytokines in the expanded tissue are scanty.

The aim of this study is to investigate the presence of inflammatory cells and secretion of inflammatory cytokines in the skin tissue expanded using the novel anisotropic hydrogel tissue expander. We believe the results will provide useful information for a better understanding on how tissue will physiologically/pathologically respond to expansion.

## 2. Materials and Methods

### 2.1. Ethical Approval

This study was approved by the Faculty of Medicine Institutional, Animal Care and Use Committee (FOM IACUC), University of Malaya. Reference number of the ethical approval is 2015-161006/DENT/R/ZR.

### 2.2. Study Design

This was an experimental in vivo study, designed to examine the effect of anisotropic hydrogel tissue expansion on inflammatory mediators and the possibility of inducing inflammatory cytokines as the body responds to the expander. This study utilized rat skin as a model.

### 2.3. Hydrogel Polymers Material

Many types of hydrogel expanders are available depending on the types of the polymer material incorporated. In this study, a methyl-methacrylate and N-vinylpyrrolidone copolymer (AMM-NVP)-based anisotropic self-inflating hydrogel was used (Oxtex, Witney, UK), at a ratio of 10:90, respectively. The polymerization reaction was initiated by azobisisobutyronitrile (0.2 wt%), and the formed gel was processed from isotropic to anisotropic as follows: The gel was subjected to high temperature at 161 °C in a thermostatically controlled hydraulic press glass chamber for about 60 min, then allowed to cool down for another 60 min under the compressor at room temperature only in one direction. Thus, the swelling behavior will only be in the direction where the force was applied, i.e., directional dependence (anisotropic).

### 2.4. Sample Selection

Skin samples were taken from Sprague Dawley rats (300 g ± 50 g). Rats were chosen for many reasons. First, they share 99% genetic similarities with humans. This will ensure that the macromolecular data will provide useful information for future clinical use. Additionally, they were large enough to make them easy to handle for surgical procedures. The age of the rats was 8 weeks, therefore eliminating any possible compounding factors resulting from growth. Moreover, biochemical materials, reagents, and biological markers are commonly available and easily accessible for those species.

### 2.5. Animal Preparation

Seven 8-week-old female Sprague-Dawley rats were used in this study. Three rats were used as controls and 4 were used for in vivo expansion of scalp tissue as described previously [8]. Briefly, the hair of the scalp was shaved, and the scalp was permanently tattooed by using indelible ink and a tattooing machine to allow accurate measurement of the degree of swelling throughout the expansion process. The rats were sedated intraperitoneal administration of 3–5 mL/Kg (body wt) ketamine (Vetoquinol UK Ltd Buckingham, UK) and 1 mL/Kg (body wt) of Diazepam (Diapine, Atlantic Laboratoreis Corporation, Bangkok, Thailand) and to improve post-operative analgesia and to minimize bleeding. A small skin incision on the frontal region was made and a subcutaneous pocket was created by blunt dissection. A sterilized tissue expander (Oxtex, Witney, UK) was implanted in the pocket at the midline of the scalp for the expanded groups. Each expander was of known dry mass and was a cylindrical device up to 30 mm in diameter and 10 mm in thickness. The surgical wound was sutured (3–0, Silk, reverse cutting needle (Unik Surgical Sutures MFG Co., New Taipei City, Taiwan) and dressed. While for the control groups, the surgical site was sutured without placing the expander. Post-operative monitoring and appropriate post-care including maintenance of temperature (20–26 °C), other environmental conditions, and analgesia were conducted according to the Institutional Animal Care and Use Committee (IACUC), UM.

Following recovery from the surgical procedures, the rats were returned to the cages when they gained consciousness. Regular visual inspection of the surgical site was performed for detection of post-operative complications. The rate of device swelling was monitored by regular visual inspection and simple measurement using a Vernier caliper (Mitutoyo Corporation, Kanagawa, Japan). Animals were monitored for a period of four weeks according to the guidelines for animal care and use (IACUC, UM), after which they were sacrificed.

### 2.6. Sample Preparation

Samples from expanded and controlled rats were taken immediately after they were sacrificed. Both control and expanded skin samples measuring 2 cm^2^ were excised and fixed in 10% formalin solution overnight at room temperature and then embedded in paraffin wax followed by micrtomoe sectioning, (thickness = 5 μm) at room temperature and mounted on polarized silane coated glass slides. The sections were then used for histology (H&E) and immunohistochemical staining. All the procedures were performed at the Oral Pathology Laboratory, Faculty of Dentistry, University Malaya.

### 2.7. Hematoxylin and Eosin (H&E) Staining

The sections were deparaffinized with two changes of xylene for 5 min each, and then rehydrated in absolute alcohol (100%) for 3 min, 95% alcohol for 3 min, and 70% alcohol for 3 min. The slides were then washed for a minute under running water. The slides were dipped in hematoxylin solution and left there for about 5 min and then washed again under running water for a minute. For differentiation, 0.5% acid alcohol was used for 10 s to remove any excess background staining and produce definitive nuclei with crisp detail. This was followed by washing under running water immediately. 2% sodium acetate buffer was used as a chromogenic staining for nucleus. The slides were dipped in the alkaline buffer four times and then placed under running water for minutes. Next, the slides were rinsed in 80% alcohol for 1 min and counter-stained in eosin solution for 2–3 min. The slides were dehydrated in 95% ethanol, and two changes in absolute ethanol for two minutes each. Clearing was performed using 3 changes of xylene for 3 min each. 

### 2.8. Immunohistochemistry

Immunohistochemical analysis of rat skin sections for pro-inflammatory cytokines IL-1, IL-6, and TNF-α was performed following the standard procedure according to the manufacturer instructions. Sections attached to glass slides were treated sequentially as follows: dewaxed and deparaffinized in 3 changes of xylene, each for 5 min (Xylene I, II, and III); rehydrated in 100% ethanol twice for 10 min each; in 95% ethanol twice for 10 min each; in PBS (two times each for 2 min); and finally in distillated water (dH_2_O). The slides were then heat-induced for antigen retrieval by citrate buffer solution which was prepared by dissolving trisodium citrate (2.94 g) in 1 L of distillated water with 500 μL of tween-20 and adjusted the pH at 6.0 by HCl. The slides were washed with deionized water and endogenous peroxidases sites were blocked by incubation in hydrogen peroxide (Dako Real peroxidase blocking solution). Next, 5% BSA (HyClone, Cat # SH30574.01) was used to block non-specific antibody binding followed by incubation with either the diluted primary antibody, rabbit monoclonal Anti-IL-1 antibody (ab124962; Abcam, Cambridge, UK), rabbit polyclonal Anti-IL-6 antibody (ab208113; Abcam, Cambridge, UK), or rabbit polyclonal Anti-TNF-α (ab6671; Abcam, Cambridge, UK) overnight at ≈90% humidity and 4 °C. Unbound primary antibodies were washed after rinsing with PBS followed by incubation with the biotinylated diluted secondary antibody which was goat anti-rabbit IgG HRP (Ab6721; Abcam, Cambridge, UK) for 2 h at room temperature and ≈90% humidity. For staining, DAB substrate kits (Dako Liquid DAB+ Substrate Chromogen System, Code K3468) were used and counterstained with hemotoxyline for 20 s. The slides were dehydrated in 95% ethanol for 10 s two times, and washed in 99% ethanol for 10 s two times. Then, the slides were treated in xylene 1 for 10 s, xylene 2 for 10 s, and xylene 3 for 10 s. The coverslips were mounted over the slides by using a Leica Biosystems mounting medium (cat # 3801732). Negative controls were kept by omission of the incubation with primary antibody.

### 2.9. Visualization of the Histological Specimens

All the slides were digitized with the Panoramic SCAN digital slide scanner (3DHISTECH, Budapest, Hungary). The histological images were subsequently analyzed by panoramic case viewer software version 1.15.3 (3DHISTECH, Budapest, Hungary) at 40× magnification.

### 2.10. Scoring of Immunohistologically Positive Stained Samples

The presence of immune reactive cells was analyzed and scored based on a semi-quantitative scoring system using panoramic case viewer software version 1.15.2 (3DHISTECH, Budapest, Hungary). Each slide was divided into nine equal targeting areas about 4 × 8 μm^2^ in size, three targeting areas for each layer of skin: epidermis, dermis, and hypoderm. The positive and negative cells were then counted at 40 times magnification. The total percentage of the positive cells was calculated by dividing the number of positive cells by the total cell number and multiplying by 100, similarly for the total negative cell percentage, and the scores were classified as 0 = negative; weak ≤ 10%; moderate = 11–50%; strong = 51–80%; and very strong ≥ 80% [19].

### 2.11. Statistical Analysis

The distribution of the data was analyzed by the Shapiro–Wilk test for the normality of the datasets, and the data were not normally distributed. Comparison between control and expanded groups was then performed by using a non-parametric independent test (Mann–Whitney U test). The data were positively skewed due to the large number of (0) values within the variance, thus the mean will not be a good representation of the data. Instead, the data were presented with the mean rank of the mentioned test [20]. The *p*-value generated from the statistical test was used to demonstrate the significant difference between the control and expanded tissues, considering significance at *p* ≤ 0.05, and with respect to a two-tailed probability distribution. The non-parametric Spearman’s correlation coefficient was used to evaluate whether the immunepositive expanded tissues were correlated with the inflammatory cells in the same particular tissue, and the (two-tailed) *p*-value was used to assess the significance of the correlation. All the analyses were performed by Statistical Package for the Social Sciences (SPSS) version 21 (IBM, Armonk, NY, USA).

## 3. Results

### 3.1. The effect of Anisotropic Hydrogel Tissue Expander on Skin Histology

**Figure 1:**
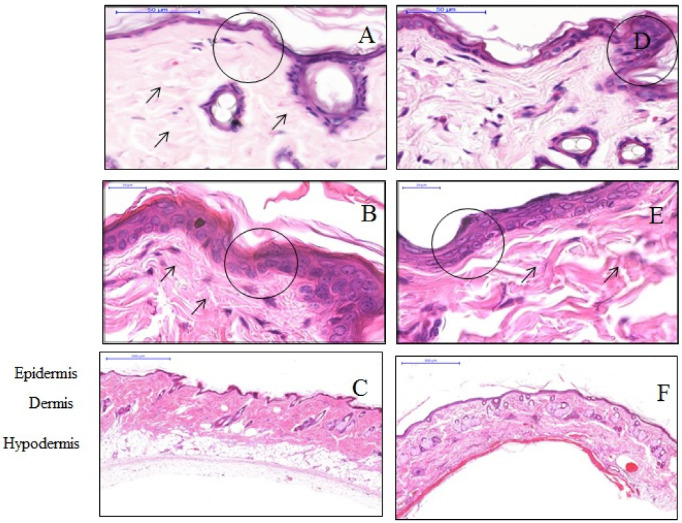
Histology of the epidermis. Representative images of control (**A**–**C**) and expanded tissues (**D**–**F**) show the difference in the thickness of the epidermis and collagen distribution in the dermis. The epidermis is thicker in control tissues that consist of 2–3 layers of cuboidal epithelial cells (shown in circle in **A**,**B**) compared to that of the expanded tissues that appear to be a single layer of flattened cells (shown in circle in **D**,**E**). The dermal collagen fibers are intact in controls (**A**) compared to that in expanded tissues, which appear to be more scattered and irregular with eosinophilic matrix (**D**). The fibers appear fragmented with variation in length and diameter in expanded tissues (indicated with arrows **E**). The hypodermal skin layer completely disappeared in the expanded tissues (**F**) in comparison to the thick cellular fat tissue in the control skin specimens (**C**).

H&E stain assessed the effect of the anisotropic hydrogel tissue expander on skin tissue histology and morphology. A standard light microscope examination of tissue specimens stained after four weeks post-expansion showed a thinner epidermis of the expanded tissues compared to that in controlled tissues. Cells in the expanded tissue were seen to be randomly distributed in a single layer. In contrast, the cells of the control tissues were distributed in two to three layers with intact regular tissue architecture. The cells of the expanded tissues were more flattened compared to the cuboidal shape of epidermis cells in control tissues. Expanded skin dermis was found to have increased thickness and a higher number of fibroblasts. In reticular dermis, the collagen fibers were arranged in an irregular manner and differed in length and diameter compared with the dermis in the control tissues. The hypodermal skin layer completely disappeared in the expanded tissues in comparison to the thick cellular fat tissue in the control skin specimens (Figure 1).

### 3.2. The effect of Anisotropic Hydrogel Tissue Expander on Inflammation

**Figure 2:**
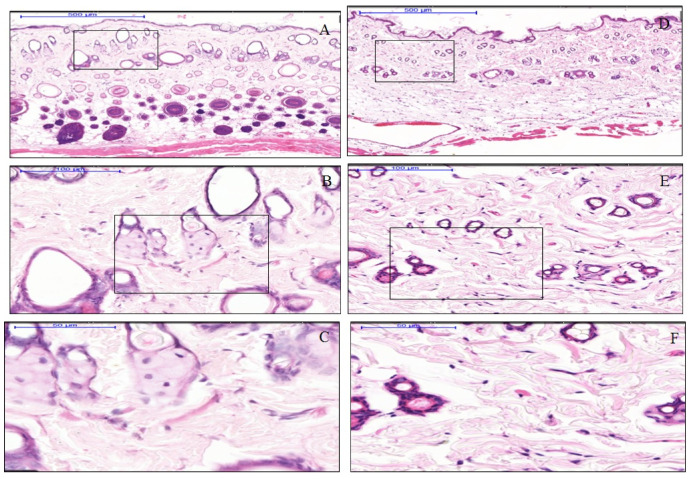
Scanning power view of hematoxylin and eosin (H&E) stained rat skin from the frontal regions. Infiltration of the inflammatory cells was not detected either in the control or in the expanded tissues. The dense band of typical lymphocytic infiltration in the superficial dermis was not present, similarly in the dermis, hypodermis expanded tissues samples (**A**–**C**), and control tissue samples (**D**–**F**). Expanded specimens (**A**–**C**) did not exhibit vascular degeneration nor prominent necrosis of individual keratinocytes.

**Figure 3:**
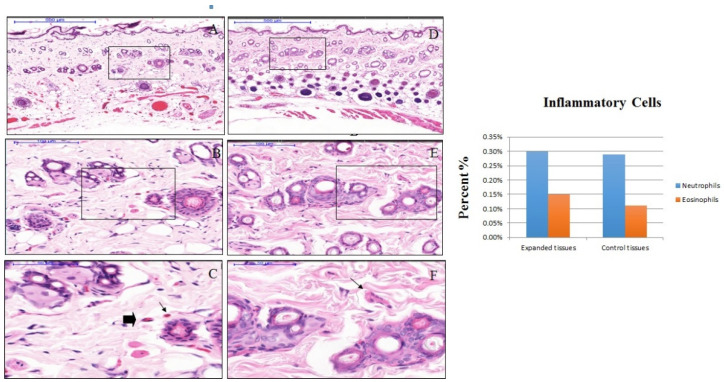
Hematoxylin and eosin (H&E) staining section of control (**D**–**F**) and expanded (**A**–**C**) rat skin. Representative serial magnifications of the dermis show isolated resident neutrophil (shown by black arrows in **C**) and eosinophil (shown by black triangle in **C**) in the connective tissue. The bar chart compares the percentage of inflammatory cells (neutrophils and eosinophils) in expanded and control tissues, with 0.30% neutrophils and 0.15% eosinophils in the expanded tissues, while 0.29% neutrophils and 0.11% eosinophils in control tissues.

The dermal papilla of the expanded tissues appeared normal and together with the dermis layer did not show the presence of an inflammatory cells band in the expanded tissues, similarly to the control tissue samples, and polymorphonuclear cells and other WBC around the dermal blood vessels were absent (Figure 2). The number of resident inflammatory cells in the dermal layer of the expanded tissue were comprised of 0.30% neutrophils and 0.15% eosinophils. While in the same layers of the control tissues, 0.29% neutrophils and 0.11% eosinophils, were present. Nonetheless, the differences the differences in the resident inflammatory cells between control and expanded tissues were not statistically significant (Figure 3).

### 3.3. The Effect of Anisotropic Hydrogel Tissue Expansion on the Expression of IL-1, IL-6, and TNF-α

**Figure 4:**
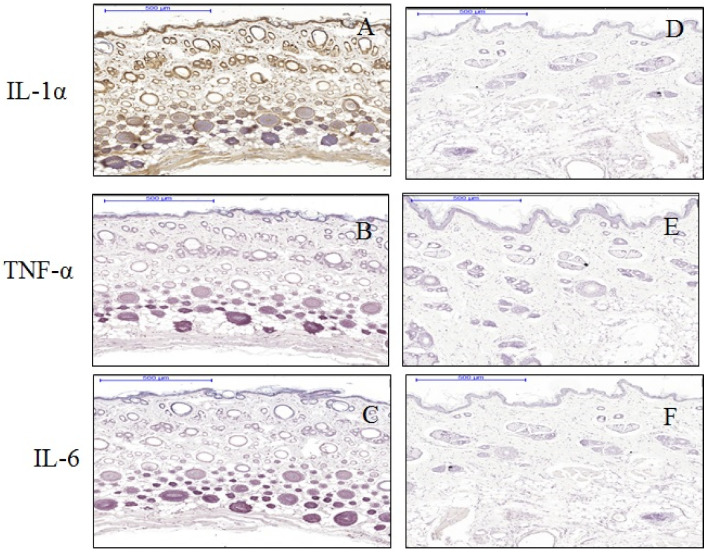
Immunohistochemical staining of IL-1 (**A**,**D**), TNF-α (**B**,**E**), and IL-6 (**C**,**F**) in expanded (**A**–**C**) and control (**D**–**F**) tissues. Expanded tissues (**A**) show strong cytoplasmic and nuclear positivity (brown color) of IL-1in the epidermis, dermis, and hypoderm compared to controls (**D**). Enhanced expression of IL-1 was detected in the nucleus of keratinocyte. Additionally, all the collagen fibers in the dermal layer were IL-1-positive compared to controls. However, expression of TNF-α and IL-6 did not show any differences between the expanded (**B**,**C**) and control tissues (**E**,**F**).

**Figure 5:**
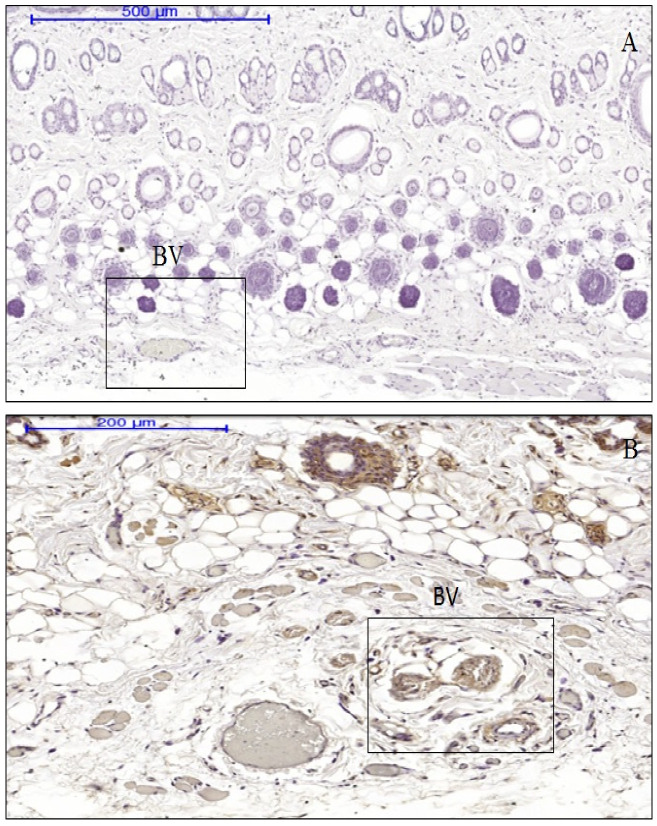
Comparison of IL-1 expression in the blood vessel regions in hypoderms. Endothelium cell in the hypoderm of the expanded tissues (**B**) showed strong positive staining for IL-1 (shown in boxes) compare to that in the controls (**A**). BV = blood vessels.

About 83% of cells (very strong scoring) were found positive for IL-1 in expanded tissue compared to none in control tissues. About 91% of keratinocytes of the epidermis (very strong scoring) were positive for cytoplasmic IL-1 in the expanded tissue. Again in the dermis of the expanded tissue, 97% (very strong scoring) cells were IL-1-positive, where IL-1 was found to be expressed both in the nucleus and cytoplasm. Most of the IL-1 positive cells in the expanded tissue were present at the glassy membrane and the medulla of the hair follicles. Notably, TNF-α and IL-6 expression were not expressed neither in the hydrogel expanded tissues nor in the control tissues (Figure 4). Enhanced IL-1 expression was observed in the cells of blood vessel cells in the expanded tissues. At the same time, endothelial cells in the hypoderm were also more IL-1 positive in the expanded tissues compared to the controls (Figure 5).

### 3.4. The Difference between Cytokine Production in Expanded and Non-Expanded Skin Tissue Samples

The production of pro-inflammatory cytokines was higher in the expanded samples (a mean rank of 14.00) compared to the controls (mean rank of 11.00). However, the differences between the mean ranks were not statistically significant (*p* = 0.071).

### 3.5. The Association between Inflammatory Cell Profiles and Cytokine Production in Immune-Positive Expanded Tissue

There was a weak negative correlation between the immune-positive expanded samples and the presence of the resident neutrophils and eosinophils associated with acute inflammation (with a Spearman’s correlation coefficient of r = −0.500). However, this negative correlation statistically was not significant (*p* = 0.667), hence, the reduction of one variable does not indicate an increase in the other.

## 4. Discussion

Clinical soft tissue expansion has become revolutionary in the last three decades where it relies on the ability of the tissue to remodel and grow when mechanical force is applied. It is an ideal way to attain extra tissue which remarkably matches in color and texture with the surrounding healthy tissue. Application of tissue expansion has reduced the common complication associated with the flap procedure to close the large defect. It was first presented by Neumann in 1957 to reconstruct injured traumatic ears using a rubber balloon filled with air [21]. Hydrogels are macromolecules with cross-link networks that are capable of taking in water and swelling in size. The network permits the passage of liquid molecules and keeps out other dissolved materials and acts as a barrier. Wide ranges of hydrophilic polymers have been quantified, but only (AMM-NVP)-based self-inflated expanders are widely and clinically used. Despite their popularity, there is a lack of understanding of how the tissue responds to them. Generally, when the skin is stretched, new skin is created and changes in tissue microstructure occur, and activation of stress-induced signaling pathways causes multiple molecules to be secreted in response to the stress to control cellular function, division, and proliferation or death. Nevertheless, immune cell activation and attraction to the surgical site are the initial responses of our body to the implanted material in general by secretion of several active biological molecules such as enzymes, lysosomes, and cytokines [16].

In the present study, we evaluated the soft tissue response to anisotropic hydrogel tissue expanders (AMM-NVP) as an inflammatory trigger of pro-inflammatory cytokines. However, our findings show that anisotropic hydrogel tissue expanders slightly change the skin morphology but had no significant effect on targeting the immunological pathways. This agrees with our hypothesis and with the clinical results of previous studies in the literature. After four weeks post-expansion, the epidermis in expanded skin samples was slightly thinner than those in controls. Moreover, an increase in dermal thickness in expanded skin was observed in this study. On the other hand, it does not induce soft tissue inflammatory responses. In fact, its effect on the immune system and inflammatory signaling is yet to be explored. Numerous studies demonstrated that tissue expansion in general would lead to pro-inflammatory cytokine secretion [16], but the specific triggers for those inflammatory cytokines and cellular origins of inflammatory mediators are still unclear. We chose to investigate IL-1α, TNF-α, and IL-6 as markers in this study because they are the primary pro-inflammatory mediators provoking and involved in the inflammatory process [22,23].

Although our current study showed no clinical manifestation of inflammation, the presence of IL-1α in the expanded tissue indicated that despite the absence of inflammation, there are other factors that could stimulate its secretion. Activation of different key modulator proteins will lead to activation of either anti- or pro-inflammatory response pathways [24,25,26,27,28]. Generally, TNF-α has always been case-dependent and linked with IL-1α. However, in this study, we did not observe that. Notably, the presence of both IL-1α and TNF-α is a strong indication of inflammation. Moreover, TNF-α in the skin is produced mainly by activation of resident dermal mast cells [29]. Those cells contain numerous sizable active TNF-α inside their granules, which can be rapidly released to the extracellular space upon degradation of the cells. Those cells are found to be the only source of TNF-α and they are located near the blood vessel. Thus, in H&E-stained sections, there was no sign of scattered mast cells, apoptotic cells, or degranulated cells. Additionally, under any inflammatory conditions, elevation of IL-6 has a direct relation with IL-1 and TNF-α, as these three cytokines together have been recognized as targets of therapeutic intervention for inflammation for many years [30]. Furthermore, studies presented information that apoptosis induces IL-6 [31]. It is reasonable that we did not detect IL-6 in immune-active cells in our expanded tissues.

As a consequence, when mechano-transduction force is applied on the skin by tissue expanders, the cellular structure and cytoskeletal network architecture are affected. Nonetheless, even though some changes are reversible after removing the expander, new tissue growth resulting from tissue creep is indeed irreversible. Dermal stretch beyond its physiological level triggers multiple different stress-induced signaling pathways [32], resulting in cellular growth activation, cytoskeletal proliferation, and increase in the neovascularization of the dermal cell layer [7]. Most of the dermis is made up of collagen [33], which makes up 70% to 90% of its dry content, giving it structure and strength [34,35]. During active tissue expansion, fibroblast cells increase in number and mitotic activity, thus increasing extracellular matrix protein and collagen deposition. Most of the data from previous studies provide information only at the macroscopic level, while further experiments are required at the cellular level to clarify the biological pathways involved in the growth process of fibroblasts in relation to tissue expansion. However, remarkable recent studies have found that IL-1 plays a crucial role in inducing and/or increasing fibroblast collagen synthesis in vitro [36,37] as IL-1 is a cross-talk signaling molecule from the epithelium cells to fibroblast cells in normal immune defense [38]. In addition, a recent study found that the expression of osteopontin (OPN), which is a pro-fibrotic molecule, is directly increased by IL-1 [39]. In the skin dermis, production of collagen by fibroblasts is down-regulated by keratinocytes via production of IL-1 [40,41]. It is noteworthy that IL-1 down-regulates angiogenesis, as the presence of IL-1 immune-positive endothelium cells in our samples indicates that the cells might produce it in response to expansion as a protective mechanism to prevent abnormal proliferation and tumor formation via the anti-angiogenic function of IL-1 [42], as tissue expansion does stimulate the formation of new blood vessels. Studies found that IL-1 directly stimulates angiogenesis via the vascular endothelium growth factor (VEGF) signaling pathway, a pathway that is crucial in normal physiological angiogenesis and wound healing. In contrast, IL-6 stimulates angiogenesis via two pathways—the NOTCH ligand Jagged pathway [43] and the angiopoietin–tie pathway—which are considered to be the main pathways involved during pathological vascular remodeling and tumor angiogenesis [44]. Thus, the absence of IL-6 in our samples may be a good indicator for physiological angiogenesis. Moreover, VEGF does result in spontaneous epithelization and collagen deposition [45]. All of the mentioned events may explain the reason behind finding IL-1α in our study.

However, some limitations should be noted. This type of tissue expander is meant to be there for four weeks as the manufacturer recommendations, however, an evaluation of inflammation at one, two, and three weeks after would be helpful to definitely conclude that the tissue expander does not induce inflammation. Moreover, it is typically noted that the larger the sample size, the higher the degree of accuracy, but this was limited by the availability of some resources.

Given the results of this in vivo study and notwithstanding its limitations, to the best of our knowledge, this study has yielded useful information on how tissue responds to novel anisotropic self-inflating hydrogel tissue expanders from a macro-molecular point of view, focusing on inflammation. However, the possible cause of cellular expression of IL-1α remains a topic of further investigation. 

## 5. Conclusions

We have shown that the anisotropic hydrogel tissue expander does not promote subclinical activation of inflammation, as the comparison between the inflammatory biomarkers of controls and expanded samples shows no difference. However, expression of IL-1 in expanded samples and the absence of inflammation indicate other factors might be the cause of its secretion. Furthermore, a study of the interaction between tissue expansion and different signaling pathways is expected to reveal how the crucial multifactorial IL-1α is secreted, and to determine its source, kinetic production, and regulation.

## References

[1] Nazir M.A. (2017). Prevalence of periodontal disease, its association with systemic diseases and prevention. Int. J. Health Sci..

[2] Atsuta A., Ayukama Y., Rondo A., Oshiro W., Matsuura Y., Furuhashi A., Tsukiyama Y., Koyano K. (2016). Soft tissue sealing around dental implants based on histological interpretation. J. Prosthodont. Res..

[3] Yilmaz H.N., Ozbilin E.O., Ustun T. (2019). The prevalence of cleft lip and palate patients: A single-center experience for 17 years. Turk. J. Orthod..

[4] Swan M.C., Bucknall D.G., Goodacre T.E., Czernuszka J.T. (2011). Synthesis and properties of a novel anisotropic self-inflating hydrogel tissue expander. Acta Biomater..

[5] Nishikubo M., Hirahara N., Gomi A., Nozoe E., Norifumi N. (2009). 3-D Analysis of Palatal Morphology Associated with Palatalized Articulation in Patients with Unilateral Cleft Lip and Palate. Oral. Sci. Int..

[6] Ebrahimi A., Ashayeri M., Rasouli H.R. (2015). Comparission of local flap and skin grafts to repair cheek skin defects. J. Cutan. Aesthet. Surg..

[7] Tepole A.B., Ploch C.J., Wong J.E. (2011). Growing skin: A computational model for skin expansion in reconstructive surgery. J. Mech. Phys. Solids.

[8] Garner J., Davidson D., Eckert G.J., Barco C.T., Park H., Park K. (2017). Reshapable polymeric hydrogel for controlled soft-tissue expansion: In vitro and in vivo evaluation. J. Control Release.

[9] Trubelja A., Bao G. (2018). Molecular mechanisms of mechanosensing and mechanotransduction in living cells. Extrem. Mech. Lett..

[10] Chen G., Cui S., You L., Li Y., Mei Y.H., Chen X. (2015). Experimental study on multi-step creep properties of rat skins. J. Mech. Behav. Biomed. Mater..

[11] Hsu C.K., Lin H.H., Harn H.I.C., Hughes M.W., Tang M.J., Yang C.C. (2018). Mechanical forces in skin disorders. J. Dermatol. Sci..

[12] Artola E.A., Trepat X., Cusachs R.P. (2018). Control of Mechanotransduction by Molecular Clutch Dynamics. Trends Cell Biol..

[13] Fleissner F., Parekh S.H. (2018). Imaging mechanotransduction: Seeing forces from molecules to cells. Curr. Opin. Biomed. Eng..

[14] Sethi K., Cram E.J., Bar R.Z. (2017). Stretch-induced actomyosin contraction in epithelial tubes: Mechanotransduction pathways for tubular homeostasis. Semin. Cell Dev. Biol..

[15] Mathieu S., Manneville J.B. (2018). Intracellular mechanics: Connecting rheology and mechanotransduction. Curr. Opin. Cell Biol..

[16] Vlahakis N., Schroeder M., Limper A., Hubmayr D. (1999). Stretch induces cytokine release by alveolar epithelial cells in vitro. Am. Physiol. Soc..

[17] Abuammah A., Maimari N., Towhidi L., Frueh J., Chooi K.Y., Warboys C., Krams R. (2018). New developments in mechanotransduction: Cross talk of the Wnt, TGF-β and Notch signalling pathways in reaction to shear stress. Curr. Opin. Biomed. Eng..

[18] Copland I.B., Post M. (2007). Stretch-activated signaling pathways responsible for early response gene expression in fetal lung epithelial cells. J. Cell. Physiol..

[19] Fedchenko N., Reifenrath J. (2014). Different approaches for interpretation and reporting of immunohistochemistry analysis results in the bone tissue. Diagn. Pathol..

[20] Milenovic Z.M. (2011). Application of mann-whitney u test in research of professional training of primary school teachers. Metodicki obzori.

[21] Nuemann N.D. (1957). The expansion of an area of skin by progressive distention of a subcutaneous balloon; use of the method for securing skin for subtotal reconstruction of the ear. Plast. Reconstr. Surg..

[22] Ahmad H., Verma S., Kumar V.L. (2018). Effect of roxithromycin on mucosal damage, oxidative stress and pro-inflammatory markers in experimental model of colitis. Inflamm. Res..

[23] Cuomo F., Coppola A., Botti C., Maione C., Forte A., Scisciola L., Cobellis G. (2018). Pro-inflammatory cytokines activate hypoxia-inducible factor 3alpha via epigenetic changes in mesenchymal stromal/stem cells. Sci. Rep..

[24] Scholz C.C., Taylor C.T. (2013). Targeting the HIF pathway in inflammation and immunity. Curr. Opin. Pharmacol..

[25] Buhrmann C., Yazdi M., Popper B., Shayan P., Goel A., Aggarwal B.B., Shakibaei M. (2018). Resveratrol Chemosensitizes TNF-beta-Induced Survival of 5-FU-Treated Colorectal Cancer Cells. Nutrients.

[26] Porta C., Larghi P., Rimoldi M., Totaro M.G., Allavena P., Mantovani A., Sica A. (2009). Cellular and molecular pathways linking inflammation and cancer. Immunobiology.

[27] Chai E.Z., Siveen K.S., Shanmugam M.K., Arfuso F., Sethi G. (2015). Analysis of the intricate relationship between chronic inflammation and cancer. Biochem. J..

[28] Jetten N., Verbruggen S., Gijbels M.J., Post M.J., Winther D.M.P., Donners M.M. (2013). Anti-inflammatory M2, but not pro-inflammatory M1 macrophages promote angiogenesis in vivo. Angiogenesis.

[29] Shirley D., McHale C., Gomez G. (2016). Resveratrol preferentially inhibits IgE-dependent PGD2 biosynthesis but enhances TNF production from human skin mast cells. Biochim. Biophys. Acta.

[30] Scheller J., Chalaris A., Arras D.S., John S.R. (2011). The pro- and anti-inflammatory properties of the cytokine interleukin-6. Biochim. Biophys. Acta.

[31] Chalaris A., Rabe B., Paliga K., Lange H., Laskay T., Fielding C.A., Scheller J. (2007). Apoptosis is a natural stimulus of IL6R shedding and contributes to the proinflammatory trans-signaling function of neutrophils. Blood.

[32] Lee T., Vaca E.E., Ledwon J.K., Bae H., Topczewska J.M., Turin S.Y., Tepole A.B. (2018). Improving tissue expansion protocols through computational modeling. J. Mech. Behav. Biomed. Mater..

[33] Aziz J., Ahmad M.F., Rahman M.T., Yahya N.A., Czernuszka J., Radzi Z. (2018). AFM analysis of collagen fibrils in expanded scalp tissue after anisotropic tissue expansion. Int. J. Biol. Macromol..

[34] Khavkin J., Ellis D.A. (2011). Aging Skin: Histology, Physiology, and Pathology. Facial Plast. Surg. Clin. N. Am..

[35] Papadopoulou A., Rizos E., Aggeli A. (2016). Rheological and Morphological Investigation of Renaturated Collagen Nanogels in Physiological-like Solution Conditions. Mater. Today Proc..

[36] Mia M.M., Boersema M., Bank R.A. (2014). Interleukin-1β Attenuates Myofibroblast Formation and Extracellular Matrix Production in Dermal and Lung Fibroblasts Exposed to Transforming Growth Factor-β1. PLoS ONE.

[37] Blum S.R., Baffet G., Théret N. (2018). Molecular and tissue alterations of collagens in fibrosis. Matrix Biol..

[38] Osei E.T., Noordhoek J.A., Hackett T.L., Spanjer A.I., Postma D.S., Timens W., Heijink I.H. (2016). Interleukin-1alpha drives the dysfunctional cross-talk of the airway epithelium and lung fibroblasts in COPD. Eur. Respir. J..

[39] Shimodaira T., Matsuda K., Uchibori T., Sugano M., Uehara T., Honda T. (2018). Upregulation of osteopontin expression via the interaction of macrophages and fibroblasts under IL-1b stimulation. Cytokine.

[40] Sun Y., Zhang J., Zhai T., Li H., Li H., Huo R., Teng J. (2017). CCN1 promotes IL-1β production in keratinocytes by activating p38 MAPK signaling in psoriasis. Sci. Rep..

[41] Harrison C.A., Gossiel F., Bullock A.J., Sun T., Blumsohn A., Neil S.M. (2006). Investigation of keratinocyte regulation of collagen I synthesis by dermal fibroblasts in a simple in vitro model. Br. J. Dermatol..

[42] Ribatti D. (2018). Interleukins as modulators of angiogenesis and anti-angiogenesis in tumors. Cytokines.

[43] Gopinathan G., Milagre C., Pearce O.M., Reynolds L.E., Dilke K.H., Leinster D.A., Balkwill F. (2015). Interleukin-6 Stimulates Defective Angiogenesis. Cancer Res..

[44] Saharinen P., Eklund L., Alitalo K. (2017). Therapeutic targeting of the angiopoietin-TIE pathway. Nat. Rev. Drug Discov..

[45] Bao P., Kodra A., Canic M.T., Golinko M.S., Ehrlich H.P., Brem H. (2009). The role of vascular endothelial growth factor in wound healing. J. Surg. Res..

